# Clinical value of sigmoid colon water exchange colonoscopy: a prospective randomized clinical trial

**DOI:** 10.1038/s41598-023-40706-4

**Published:** 2023-08-22

**Authors:** Tian-Xiao Jiao, Yang Hu, Shi-Bin Guo

**Affiliations:** 1 Department of Gastroenterology, The First Affiliated Hospital of Dalian Medical University, 222 Zhongshan Road, Dalian, 116011 Liaoning Province People’s Republic of China; 2 Department of Gastroenterology, Dalian Friendship Hospital, Dalian, 116011 Liaoning People’s Republic of China

**Keywords:** Gastroenterology, Colonoscopy

## Abstract

This prospective randomized controlled trial investigated the clinical value of sigmoid colon water exchange (SWE) colonoscopy by comparing it with air insufflation (AI) colonoscopy in terms of the patient’s pain score, insertion time, and screening quality. Consecutive patients who underwent colonoscopy without sedation were randomized into an AI group (n = 267) or an SWE group (n = 255). Patient characteristics, history of abdominal or pelvic surgery, maximum pain score, insertion time, cecal intubation rate, polyp detection rate, and the need for maneuvers were recorded. There was no significant between-group difference in insertion time, cecal intubation rate, assisted maneuvers (abdominal pressure, changing patients’ position), or polyp detection rate (*P* > 0.05). The mean maximum pain score was significantly lower in the SWE group than in the AI group. (3.57 ± 2.01 vs. 4.69 ± 1.83, *P* < 0.001). For patients with a history of abdominal or pelvic surgery and those who were overweight (body mass index > 24), the maximum pain scores were lower in the SWE group than in the AI group (3.67 ± 1.95 vs. 4.88 ± 1.80, *P* < 0.001; 3.40 ± 1.96 vs. 4.79 ± 1.97, *P* < 0.001, respectively). SWE colonoscopy can significantly reduce abdominal pain with non-inferior screening quality and does not increase insertion time.

*Trial registration number*: ChiCTR2200059057 (date April 23, 2022).

## Introduction

Colonoscopy is the most important examination in the diagnosis, follow-up, and treatment of colorectal diseases^1^. Air is used for conventional colonoscopy to inflate the lumen of the colon, permit visualization, and allow passage of instruments^2^. However, excessive air may result in patient discomfort by causing angulations and formation of loops. Some patients refuse colonoscopy because of poor ability to tolerate the procedure, which may lead to delayed diagnosis and treatment. Although colonoscopy under sedation can alleviate discomfort, some patients are not suitable for sedation for various reasons, including potential risk factors and increased medical costs^3^. Water-aided methods have been used to replace air insufflation (AI) during the insertion phase to minimize pain and improve ease of insertion^4^. Water-aided colonoscopy is classified as water immersion (WI) and water exchange (WE). When using the WI method, water is infused to facilitate cecal intubation and aspirated during withdrawal, whereas when using the WE method, the infused water is removed during insertion to allow progression in clear water^5,6^. It has been reported that compared with WI and AI, WE can reduce pain during insertion and improve the adenoma detection rate (ADR) but is more time-consuming^4,7^. The prolonged insertion time when using the WE method is thought to be a major obstacle to its widespread application^8,9^.

To overcome the shortcomings of the WE method, we have modified the procedure such that water exchange is performed only in the sigmoid colon. This study aimed to determine whether sigmoid colon water exchange (SWE) can reduce abdominal pain during colonoscopy and to assess its effects on secondary outcomes, including insertion time, cecal intubation rate, polyp detection rate (PDR), and the need for assisted maneuvers, such as abdominal pressure, application of an endoscope stiffener, and changing the position of the patient.

## Methods

This prospective randomized controlled trial was approved by the Ethics Committee of the First Affiliated Hospital of Dalian Medical University (approval number PJ-KY-2021-66) and performed at this hospital in accordance with the Declaration of Helsinki and local legislation. Written informed consent was obtained from the patients or their relatives at the time of enrollment.

A total of 547 consecutive patients underwent colonoscopy without sedation between May 2021 and December 2021 and were considered for enrollment in the study. The following exclusion criteria were applied: severe benign or malignant intestinal stenosis; severe cardiopulmonary disease or other contraindication to colonoscopy; aged younger than 18 years or older than 80 years; pregnancy or lactation; psychiatric illness; poor bowel preparation (Boston bowel preparation score < 6); history of colorectal surgery; inflammatory bowel disease, polyposis syndrome, or ischemic colitis; and unwillingness to participate.

The primary outcome in this study was the pain score during colonoscopy. A review of similar studies showed that the mean pain score was 2.5 ± 2.5 in the study groups and 3.4 ± 2.8 in the control groups (α = 0.05, bilateral test, 1−β = 0.9)^10^. Allowing for allocation of patients to our study group and our control group in a ratio of 1:1, the minimum sample size required for each group in the present study was calculated to be 184 cases. Considering factors such as loss to follow-up, 255 cases were enrolled in the study group and 267 in the control group.

Patient data, including sex, age, body mass index, previous abdominal or pelvic surgery, concomitant diseases (e.g., hypertension, diabetes mellitus, and coronary artery disease), and reasons for colonoscopy were recorded before the procedure. The patients were divided into an SWE group and an AI group using a computer-generated randomization list according to the order in which they were enrolled. Sealed envelopes containing the group allocation were opened by the endoscopist immediately before the procedure. The endoscopist had performed more than 6,000 colonoscopies, including 300 SWE procedures, before the start of the study. Upon arrival at the cecum or starting withdrawal, an assistant who was blinded to group allocation asked the patient about their pain level. Procedural data were also recorded, including whether the cecum was reached, cecal insertion time, polyp detection, abdominal pressure, changes in position, application of an endoscope stiffener, withdrawal time, and Boston bowel preparation score.

Polyethylene glycol solution was administered for bowel preparation in the standard split dose (1 L in the evening of the day before the procedure and 2 L in the morning of the day of the procedure). Colonoscopy was performed under electrocardiographic monitoring and started with the patient in the left lateral position. Tetracaine was administered before starting the procedure. All procedures were performed by the same endoscopist using a standard adult variable stiffness colonoscope (CF-H290I; Olympus, Tokyo, Japan). In the SWE group, after reaching the rectum, water was infused by a foot switch-controlled water pump (OFP Endoscopic Flushing Pump; Olympus) through the biopsy channel. Air, residual fecal debris, and infused water were removed by suction during insertion to minimize distention of the lumen of the colon^11^. After arriving at the descending colon, the procedure was the same as for AI. In the AI group, the lumen of colon was inflated by air, and colonoscopy was performed in the usual manner. Neither CO_2_ nor antispasmodic agents were used in both groups. In both groups, abdominal pressure, a change in the patients’ position, and stiffening of the colonoscope were implemented when needed. Washing of the mucosa was allowed when necessary and was predominantly during withdrawal, and interventions such as biopsy and polypectomy were performed during the withdrawal phase.

Insertion time was defined as the time taken for passage of the colonoscope from the rectum to the cecum. Cecal intubation was defined as arrival at the cecum with adequate visualization of the appendiceal orifice^12^. Pain was assessed using the visual analog scale score (0 = absence of pain, 10 = worst imaginable pain). A minimum withdrawal time of 6 min was required^13^.

The primary outcome was the degree of abdominal pain during insertion, and secondary outcomes included insertion time, cecal intubation rate, PDR, and need for maneuvers (e.g., abdominal pressure, application of an endoscope stiffener, and changing the position of the patient). All procedural data were recorded.

### Statistical analysis

Continuous variables are expressed as the mean ± standard deviation and were compared between groups using the Student’s* t*-test. Categorical variables are expressed as percentages and were compared using the chi-squared test or Fisher’s exact test. The statistical analysis was performed using SPSS version 25.0 software (IBM Corp., Armonk, NY, USA). A *P *value < 0.05 was considered statistically significant.

### Ethical approval

The study was conducted in compliance with the Helsinki Declaration and in accordance with local legislation, was approved by the Ethics Committee of First Affiliated Hospital of Dalian Medical University. (Ethics References No: PJ-KY-2021-66). Written informed consent was obtained from all of the patients or their relatives before the procedure.

## Results

**Figure 1:**
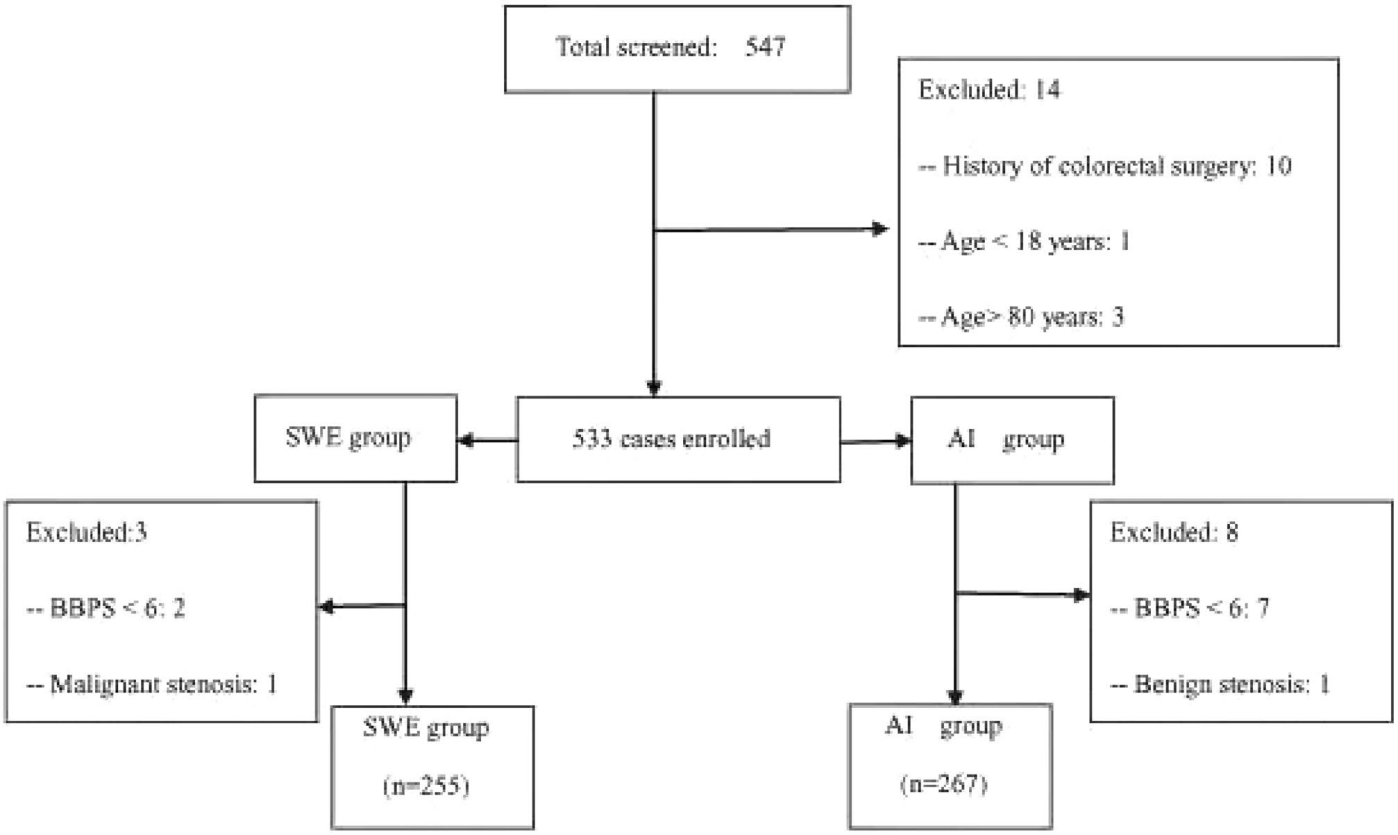
Patient enrollment process and outcomes. *AI* Air insufflation; *BBPS* Boston bowel preparation score; *SWE* Sigmoid water exchange.

After application of the exclusion criteria, 522 patients (AI group, n = 267; SWE group, n = 255) were enrolled in the study between May 2021 and December 2021 (Fig. 1). There were 314 male patients (60.15%) and 208 female patients (39.85%), giving a male-to-female ratio of 1.51:1. The mean patient age was 49.42 ± 13.55 years (range 18–78).

The demographic, anthropometric, and clinical characteristics of the 522 patients are presented in Table 1. There were no statistically significant between-group differences.

**Table 1:** Comparison of patient demographic, anthropometric, and clinical characteristics between the two study groups.

Item	SWE group (n = 255)	AI group (n = 267)	t or χ^2^ or z value	*P* value
Age	49.79 ± 13.28	49.06 ± 13.81	0.614	0.54
Sex			1.397	0.237
Male	160 (62.75%)	154 (57.68%)		
Female	95 (37.25%)	113 (42.32%)		
BMI (kg/m^2^)	24.53 ± 3.26	24.08 ± 3.46	1.544	0.123
Underweight (< 18)	9 (3.53%)	8 (3.00%)	0.118	0.732
Overweight (> 24)	101 (39.61%)	92 (34.46%)	1.485	0.223
History of hypertension	31 (12.16%)	30 (11.24%)	0.107	0.743
History of diabetes	11 (4.31%)	9 (3.37%)	0.315	0.575
History of coronary disease	8 (3.14%)	9 (3.37%)	0.023	0.881
History of pelvic surgery	30 (11.76%)	27 (10.19%)	0.366	0.545
History of abdominal surgery	29 (11.37%)	46 (17.36%)	3.635	0.057
Indication				
Abdominal discomfort	121 (47.45%)	111 (41.57%)	1.825	0.177
Diarrhea	38 (14.90%)	40 (14.98%)	0.001	0.98
Blood in stool	9 (3.53%)	12 (4.49%)	0.315	0.575
Weight loss	7 (2.75%)	10 (3.75%)	0.414	0.52
Follow-up of polypectomy	22 (8.63%)	28 (10.49%)	0.521	0.471
Medical screening	58 (22.75%)	66 (24.72%)	0.281	0.596
BBPS	8 (8,9)	8 (8,9)	− 0.191	0.849

*AI* Air insufflation; *BBPS* Boston bowel preparation score; *BMI* Body mass index; *SWE* Sigmoid colon water exchange.

### Primary outcome

**Figure 2:**
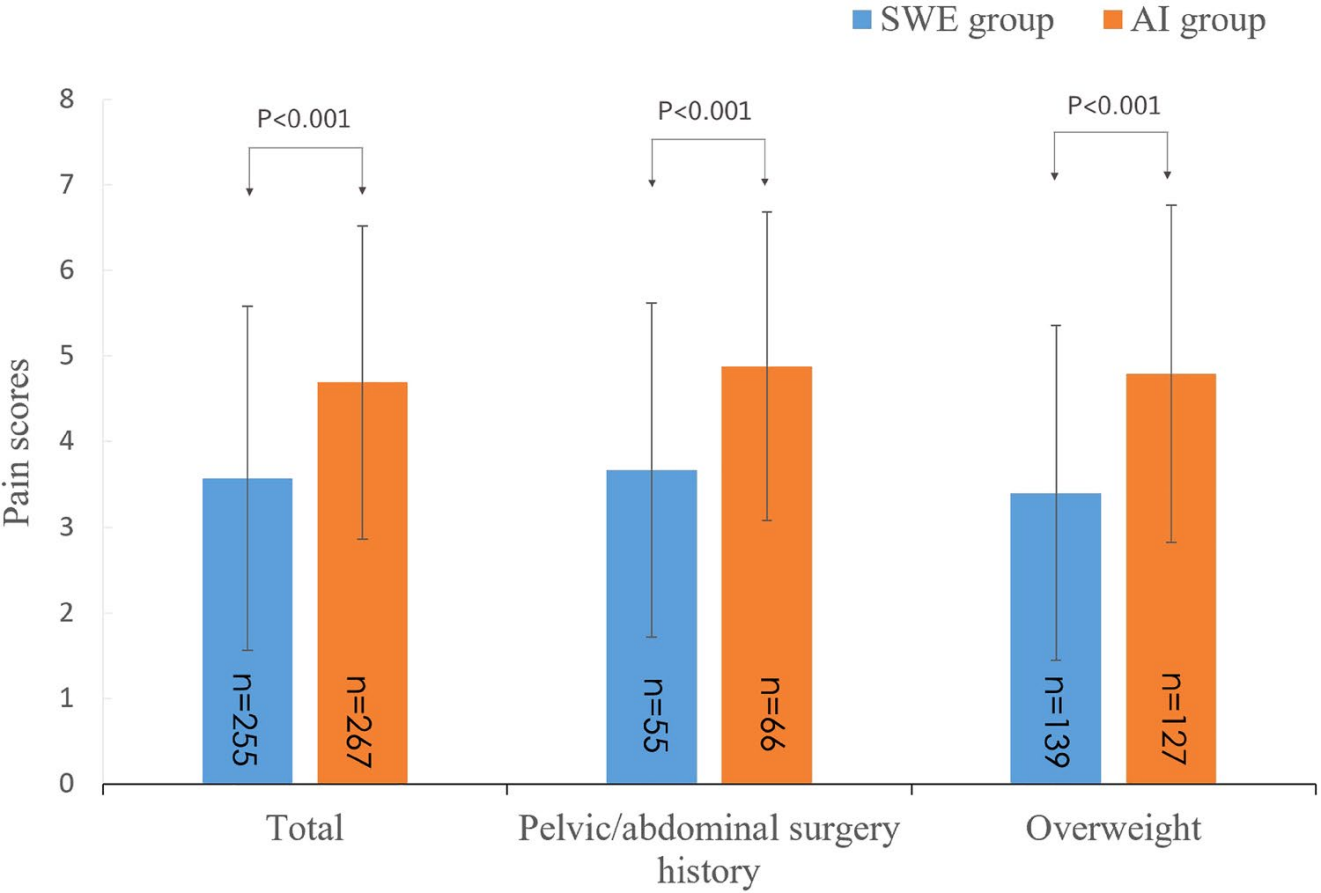
Comparison of pain scores between the two study groups. (*SWE* Sigmoid colon water exchange group, *AI* Air insufflation group).

The overall maximum pain score was significantly lower in the SWE group than in the AI group (3.57 ± 2.01 vs. 4.69 ± 1.83, *P* < 0.001). The proportion of colonoscopies that were painless was significantly higher in the SWE group (9.80% vs. 1.50%, *P* < 0.05). For patients with a history of abdominal or pelvic surgery, the maximum pain score was significantly lower in the SWE group than in the AI group (3.67 ± 1.95 vs. 4.88 ± 1.80, *P* < 0.001). For overweight patients (BMI > 24), the maximum pain score was also significantly lower in the SWE group than in the AI group (3.40 ± 1.96 vs. 4.79 ± 1.97, *P* < 0.001); for underweight patients (BMI < 18), there was no significant between-group difference in the maximum pain score (3.89 ± 2.62 vs. 3.88 ± 1.25, *P* = 0.989) (Table 2) (Fig. 2).

**Table 2:** Comparisons of pain scores between the two study groups.

Pain scores	SWE group	AI group	t value	*P* value
	(n = 255)	(n = 267)		
Total	3.57 ± 2.01	4.69 ± 1.83	− 6.694	< 0.001
Pelvic/abdominal surgery history	3.67 ± 1.95	4.88 ± 1.80	− 3.622	< 0.001
Underweight (BMI < 18 kg/m^2^)	3.89 ± 2.62	3.88 ± 1.25	0.014	0.989
Overweight (BMI > 24 kg/m^2^)	3.40 ± 1.96	4.79 ± 1.97	− 4.942	< 0.001

*AI* Air insufflation; *BMI* Body mass index; *SWE* Sigmoid colon water exchange.

### Secondary outcomes

There was no statistically significant difference in insertion time between the SWE and AI groups (5.06 ± 2.41 vs. 4.94 ± 2.41, *P* > 0.05) nor in the cecal intubation rate, frequency of assisted maneuvers (abdominal pressure, changing the position of the patient), or PDR (*P* > 0.05). However, the endoscope stiffener application rate was significantly lower in the SWE group than in the AI group (55.69% vs. 64.42%, *P* = 0.042) (Table 3).

**Table 3:** Comparison of outcomes between the two study groups.

Item	SWE group (n = 255)	AI group (n = 267)	t or χ^2^ or z value	*P* value
Reach the cecum	254 (99.61%)	265 (99.25%)	< 0.001	> 0.999
Insertion time of (min)	5.06 ± 2.41	4.94 ± 2.41	0.56	0.576
Total number of polyps	385(55.80%)	306 (44.20%)	1.095	0.274
PDR	132 (51.76%)	131 (49.06%)	0.381	0.537
Withdraw time (min)				
With Polypectomy	16.86 ± 8.43	15.34 ± 7.48	1.203	0.231
Without Polypectomy	8.39 ± 3.46	8.46 ± 2.68	− 0.228	0.82
Change posture	13 (5.10%)	11 (4.12%)	0.285	0.594
Press the abdomen	56 (21.96%)	61 (22.85%)	0.059	0.808
Application of endoscope stiffener	142 (55.69%)	172 (64.42%)	4.15	0.042

*AI* Air insufflation; *PDR* Polyp detection rate; *SWE* Sigmoid colon water exchange.

## Discussion

At present, colonoscopy is an almost indispensable cancer screening tool with the potential to reduce the morbidity and mortality of colorectal cancer^14^ by allowing detection and resection of precancerous lesions and malignant lesions in the early stages^15^. However, traditional colonoscopy requires air insufflation, which may cause a high level of discomfort during the procedure^2^. It has been reported that water-assisted methods, especially the WE method, can reduce pain markedly during insertion^5,16,17^, improve ease of insertion^18,19^ and increase the ADR^16^. In this study, we found that abdominal pain during insertion was significantly less severe in non-sedated patients who underwent SWE colonoscopy than in those who underwent AI colonoscopy, which is consistent with previous reports^17^. Difficult colonoscopy related to angulation is usually observed in thin patients and those with a history of abdominal or pelvic surgery^20^, while issues related to redundancy or excessive looping are often observed in patients with central obesity or severe constipation^21^. In our study, overweight patients and patients with a history of abdominal or pelvic surgery in the SWE group experienced less pain during insertion, possibly because when the patient is lying on their left side, the sigmoid colon can be weighted down when filled with water, which may straighten the sigmoid colon and make tight angles less acute. It is well known that 90% of pain during colonoscopy is caused by loops in the sigmoid colon. SWE may attenuate pain during colonoscopy by preventing formation of these loops. Furthermore, unlike AI, the colon is not lengthened by use of water. Moreover, water acts as a lubricant, aiding the passage of instruments, and can decrease colonic spasm. Liu et al.^22^ found that minimal WE, that is, a modified WE method in which water is infused only into the left colon by maintaining pressure on the air–water valve, could reduce abdominal pain by reducing loop formation. This could be an alternative approach for centers without a water pump.

Successful cecal intubation is one of the most important outcomes in colonoscopy, and the recommended target cecal intubation rate is 95%^13^. In our study, the cecal intubation rate was more than 99% in both groups, so no advantage of SWE in terms of improving ease of insertion was shown. This may reflect our relatively small sample size. However, all the colonoscopies in this study were performed by the same endoscopist, which would have removed any bias caused by endoscopists with different levels of experience.

Previous studies have shown that insertion is more time consuming with WE than with AI^4^ because of the extra time needed to suction dirty water and replace it with clean water^23^. In our study, WE was performed only at the sigmoid colon to overcome this shortcoming, and there was no statistically significant difference in insertion time between the two groups. We also found that SWE significantly alleviated pain and was no more time consuming than conventional AI colonoscopy. Therefore, we consider that the time factor need not be a consideration with SWE.

The ADR is one of the most important aspects of colonoscopy because it is an independent risk factor for interval colorectal cancer^24,25^. In our study, some patients did not undergo polypectomy and pathological examination, and PDR was used as a quality indicator for colonoscopy in these patients, given that it can estimate the ADR in screening and symptomatic populations^26,27^. Previous studies have shown that the ADR is higher for WE than for AI^28^, which is partly attributed to the longer withdrawal time and better bowel preparation during a WE procedure^21,29^. Furthermore, the underwater view can make polyps that are hidden behind a fold or an angulation easier to detect^4^. Moreover, when using the water-assisted method, the lumen is not fully distended, so polyps are not flattened and more easily detected than when using the air infusion method. However, there was no significant between-group difference in the PDR in our study, which we attribute to WE being performed only in the sigmoid colon. Furthermore, bowel preparation was good overall in the two groups, and the withdrawal time was more than 6 min in all patients.

Up to 2 L of water is infused into the patient’s colon during water-assisted colonoscopy, which raises concerns about safety^21^. In our study, WE was performed only in the sigmoid colon with infusion of no more than 250 mL of sterile water, and there was no change in cardiac rhythm, blood pressure, or oxygen saturation before, during, or after the examination.

This study has some limitations. First, all patients were enrolled from a single center and the sample size was relatively small. Second, the endoscopist could not be blinded, which may have introduced a degree of bias. The method used to record the patient’s pain may also have been a source of bias. Further studies in large sample sizes are required to confirm the clinical value of SWE colonoscopy without sedation.

In summary, SWE colonoscopy can significantly reduce abdominal pain with non-inferior screening quality. Furthermore, it does not increase insertion time and reduces the need for application of an endoscope stiffener.

## Data Availability

The datasets used and/or analyzed during the current study are available from the corresponding author on reasonable request.
